# A Contract-based System for Physician Practice in Their Home Region in Japan: Follow-up Data of Graduates from Jichi Medical University (1978-2021)

**DOI:** 10.31662/jmaj.2026-0078

**Published:** 2026-08-28

**Authors:** Jun Watanabe, Daisuke Matsubara, Akihisa Nakamura, Kazuhiko Kotani

**Affiliations:** 1 Division of Community and Family Medicine, Center for Community Medicine, Jichi Medical University, Shimotsuke, Tochigi, Japan

**Keywords:** career path, medically underserved area, physician distribution, primary care, rural health

## Abstract

**Introduction:**

Jichi Medical University (JMU) was founded in Japan in 1972 to provide tuition-free medical education in exchange for a nine-year service commitment in graduates’ home prefectures, with the mission of training physicians for rural practice. The long-term outcomes of this model remain incompletely characterized. We aimed to examine the long-term outcomes of JMU graduates.

**Methods:**

This descriptive, retrospective, repeated cross-sectional study followed JMU graduates from 1978 onward, with assessments in 2011, 2016, and 2021. The primary outcome was the proportion of graduates practicing in their home prefecture. Secondary outcomes were the proportions practicing in medically underserved areas, defined as municipalities designated as physician-shortage areas, and in primary care-related specialties (defined as internal medicine, surgery, pediatrics, community/rural/general medicine). Outcomes were reported separately for the during-obligation and post-obligation periods; for secondary outcomes, the during-obligation period excluded residency (postgraduate years 1-2 training). Proportions are reported with Wilson score 95% confidence intervals (CIs).

**Results:**

Among 4,610 graduates, we analyzed data from 4,534 individuals, of whom 19.0% were women. The proportion practicing in the home prefecture during the obligation period was 97.2% (4,408/4,534; 95% CI, 96.7-97.7) for up to nine years after graduation. In 2021, after the obligation period, the proportion currently practicing in the home prefecture was 71.0% (2,493/3,511; 95% CI, 69.5-72.5). The proportion of graduates practicing in medically underserved areas was 24.8% (195/787; 95% CI, 21.9-27.9) during the obligation period and 12.1% (426/3,511; 95% CI, 11.1-13.3) after the obligation period. The proportion of respondents reporting primary care-related specialties was 78.5% during the obligation period and 62.0% after the obligation period.

**Conclusions:**

The proportions of JMU graduates practicing in their home prefecture and in medically underserved areas remained stable across survey years. Additionally, JMU graduates appeared to work largely in primary care-related specialties.

## Introduction

Physician shortages are a persistent challenge in rural medical care worldwide ^(1)^. To address the geographic maldistribution of physicians, countries have generally used two models of rural workforce programs: education-based rural training programs without a service obligation and a contract-based system with a return-of-service obligation ^(2)^. The latter model provides substantial financial support for medical education in exchange for a defined return-of-service obligation in medically underserved areas, as seen in Japan ^(3), (4)^.

In Japan, this contract-based approach includes Jichi Medical University (JMU) and the regional quota program ^(4)^. JMU was established in 1972 by the national government in collaboration with all 47 prefectures to improve medical care in medically underserved areas ^(3)^. Over 50 years, nearly 5,000 physicians have graduated since JMU was founded ^(3)^. In return for tuition support, graduates are obligated to work in their home prefecture for 9 years, which generally includes postgraduate training followed by several years of prefecture-assigned service ^(3)^. Graduates who complete the obligation have their education costs waived ^(3)^. The training includes primary care specialties that are useful for rural practice ^(4)^.

In Japan, evidence on very long-term outcomes of rural physician systems remains limited, particularly for newer contract-based schemes such as the regional quota program, where post-obligation outcomes are still unknown ^(3)^. The workforce outcomes of JMU graduates were previously reported through 2006, but whether the JMU system has maintained comparably high rural service levels over the subsequent two decades of changing health-system and societal contexts remains undetermined ^(3)^. The present study aimed to investigate the long-term outcomes of the JMU graduates, with particular attention to practicing in their home prefecture, medically underserved areas, and primary care-related specialties.

## Materials and Methods

This was a descriptive, retrospective, repeated cross-sectional study using data from 2011, 2016, and 2021. This study was approved by the Ethics Review Board at JMU (approval No. Clin 25-039). All procedures were conducted anonymously in accordance with the Declaration of Helsinki ^(5)^.

### Subjects and outcomes

We used data on JMU graduates from 1978 to 2021. Graduates who had died or did not pass the Japanese National Medical Licensing Examination were excluded from the study. The primary outcome was the proportion of graduates practicing in their home prefecture during the obligation period and at the survey time point. Practice in the home prefecture during the obligation period was assessed from graduation through the survey year for graduates surveyed during the obligation period and across the obligation period for graduates surveyed after the obligation period. Practice in the home prefecture at the survey time point was defined as the graduate’s current workplace at the time of each survey. Secondary outcomes were the proportions practicing in medically underserved areas and primary care-related specialties at each survey time point.

Medically underserved areas were defined using a fixed designation list based on the 2021 prefecture-designated physician-shortage municipalities within Japan’s physician maldistribution policy framework ^(6), (7)^. In this framework, the Ministry of Health, Labor and Welfare provides a national physician maldistribution index and a common thresholding approach ^(6), (7)^. Prefectures then designate physician-shortage areas within the physician securing plan, which is periodically revised. Because graduate placements do not rely on uniform “rural” definition ^(3)^, we used this nationally standardized designation as a policy-relevant proxy for underserved areas. Because this policy-based designation was operationalized from 2020 onward and comparable historical lists were not consistently available for earlier years, we applied the 2021 designation list uniformly to all waves (2011/2016/2021) ^(6), (7)^. Primary care-related specialties were defined as internal medicine, surgery, pediatrics, and community/rural/general medicine based on self-declared specialties according to a previous Japanese study ^(3)^ and local workforce context. Outcomes were reported separately for the during-obligation (postgraduate years [PGYs] 1-9) and post-obligation (PGY ≥10) periods; for secondary outcomes, the during-obligation period excluded residency (PGY 1-2 training). Work locations were ascertained annually through a postal survey conducted by the Alumni Association, which mailed a letter to each graduate and recorded the location based on the graduates’ reports. Specialties were also recorded on the basis of self-reports.

### Statistical analysis

Categorical variables are presented as numbers (percentages). We quantified the number and proportion of missing observations for each wave and outcome. In the primary analysis, individuals with missing outcome information were classified as not having the outcome and were included in the denominator. In a best-case sensitivity analysis, we reclassified missing outcome information as outcome-present by counting these individuals in the numerator while keeping the same denominator. Data management was performed using Microsoft Excel (ver. 16.103). Two-sided 95% confidence intervals (CIs) for proportions were calculated using the Wilson score method in R software version 4.5.2 (R Foundation for Statistical Computing, Vienna, Austria).

### Results

Of 4,610 graduates identified in 2021, 76 who had died were excluded, leaving 4,534 eligible graduates for analysis; 19.0% were women. In 2016 and 2011, 61 and 40 graduates, respectively, who had died or were unlicensed at the time of assessment were excluded, leaving 3,963 and 3,441 eligible graduates. Analyses of work location, including current practice in the home prefecture and practice in medically underserved areas, included 44/4,534 graduates (97.2%) in 2021, 3,861/3,963 (97.4%) in 2016, and 3,334/3,441 (96.9%) in 2011.

Most graduates fulfilled their 9-year service obligation by working in their home prefecture, with rates of 4,408/4,534 (97.2% [95% CI, 96.7-97.7]) in 2021, 3,861/3,963 (97.4% [95% CI, 96.9-97.9]) in 2016, and 3,334/3,441 (96.9% [95% CI, 96.3-97.4]) in 2011 (Table 1). After completing the obligation, the proportion currently practicing in the home prefecture was consistent across waves: 2,493/3,511 (71.0% [95% CI, 69.5-72.5]) in 2021, 2,138/3,028 (70.6% [95% CI, 69.0-72.2]) in 2016, and 1,836/2,548 (72.1% [95% CI, 70.3-73.8]) in 2011 up to the postgraduate year 44 (Table 2).

**Table 1.** The Data of All Graduates.

Year	2011	% (95% CI)*	2016	% (95% CI)*	2021	% (95% CI)*
Total graduates, n	3,481		4,024		4,610	
Excluded^†^, n	40		61		76	
Eligible graduates, n	3,441		3,963		4,534	
Practice in their prefecture during obligation, n^‡^	3,334	96.9 (96.3-97.4)	3,861	97.4 (96.9-97.9)	4,408	97.2 (96.7-97.7)
Current practice in their prefecture, n^‡^	2,506	72.8 (71.3-74.3)	2,830	71.4 (70.0-72.8)	3,262	71.9 (70.6-73.2)
Practice in medically underserved areas, n^‡^	524	15.2 (14.1-16.5)	581	14.7 (13.6-15.8)	634	14.0 (13.0-15.0)
Missing data, n	107	3.2	102	2.6	126	2.6
Primary care-related specialties, n^‡^	2,151	62.5 (60.9-64.1)	2,428	61.3 (59.7-62.8)	2,796	61.7 (60.2-63.1)
Missing data, n	647	18.8	582	14.7	696	15.4

CI: confidence interval. *Percentages were calculated using eligible graduates with non-missing outcome data as denominators for each row. 95% CIs for proportions were calculated using the Wilson method. ^†^Deaths or unlicensed at assessment. ^‡^Practice in home prefecture during obligation was defined over the relevant period. For graduates surveyed during the obligation period, this period was from graduation to the survey year; for those surveyed after the obligation period, it was the obligation period. Current practice in the home prefecture, practice in medically underserved areas, and primary care-related specialties were assessed at the survey time point.

**Table 2.** Graduates After Completing the Obligatory Service Period.

Year	2011	% (95% CI)*	2016	% (95% CI)*	2021	% (95% CI)*
Eligible graduates, n	2,548		3,028		3,511	
Current practice in their prefecture, n^†^	1,836	72.1 (70.3-73.8)	2,138	70.6 (69.0-72.2)	2,493	71.0 (69.5-72.5)
Practice in medically underserved areas, n^†^	329	12.9 (11.7-14.3)	376	12.4 (11.3-13.6)	426	12.1 (11.1-13.3)
Missing data, n	25	3.6	20	2.8	18	2.3
Primary care-related specialties, n^†^	1,635	64.2 (62.3-66.0)	1,903	62.8 (61.1-64.6)	2,178	62.0 (60.4-63.6)
Missing data, n	401	15.7	496	16.4	631	18.0

CI: confidence interval. *Percentages were calculated using eligible graduates with non-missing outcome data as denominators for each row. 95% CIs for proportions were calculated using the Wilson method. ^†^Current practice in the home prefecture, practice in medically underserved areas, and primary care-related specialties were assessed at the survey time point.

The proportion of JMU graduates practicing in medically underserved areas was 195/787 (24.8% [95% CI, 21.9-27.9]) in 2021, 186/712 (26.1% [95% CI, 23-29.5]) in 2016, 180/695 (25.9% [95% CI, 22.8-29.3]) in 2011 during the obligation period (Table 3), 426/3,511 (12.1% [95% CI, 11.1-13.3]) in 2021, 376/3,028 (12.4% [95% CI, 11.3-13.6]) in 2016, and 329/2,548 (12.9% [95% CI, 11.7-14.3]) in 2011 after the obligation period. The proportion of graduates with primary care-related specialties was 618/787 (78.5% [95% CI, 75.5-81.3]) in 2021, 525/712 (73.7% [95% CI, 70.4-76.8]) in 2016, 516/695 (74.2% [95% CI, 70.9-77.4]) in 2011 during the obligation period, 2,178/3,511 (62.0% [95% CI, 60.4-63.6]) in 2021, 1,903/3,028 (62.8% [95% CI, 61.1-64.6]) in 2016, and 1,635/2,548 (64.2% [95% CI, 62.3-66.0]) in 2011 after the obligation period. In medically underserved areas, the proportion of graduates practicing in primary care-related specialties was high during the obligated service period (166/195 [85.1%, 95% CI, 79.5-89.4] in 2021, 163/186 [87.6%, 95% CI, 82.1-91.6] in 2016, and 157/180 [87.2%, 95% CI, 81.6-91.3] in 2011) and remained substantial after the obligation (322/426 [75.6%, 95% CI, 71.3-79.4] in 2021, 280/376 [74.5%, 95% CI, 69.8-78.6] in 2016, and 252/329 [76.6%, 95% CI, 71.7-80.8] in 2011).

**Table 3.** Graduates During the Obligatory Service Period Excluding Postgraduate Year 1-2 Training.

Year	2011	% (95% CI)*	2016	% (95% CI)*	2021	% (95% CI)*
Eligible graduates, n	695		712		787	
Current practice in their prefecture, n^†^	670	96.4 (94.7-97.6)	692	97.2 (95.7-98.2)	769	97.7 (96.4-98.5)
Practice in medically underserved areas, n^†^	180	25.9 (22.8-29.3)	186	26.1 (23-29.5)	195	24.8 (21.9-27.9)
Missing data, n	92	3.6	108	3.6	119	3.9
Primary care-related specialties, n^†^	516	74.2 (70.9-77.4)	525	73.7 (70.4-76.8)	618	78.5 (75.5-81.3)
Missing data, n	60	8.6	86	12.1	65	8.3

CI: confidence interval. *Percentages were calculated using eligible graduates with non-missing outcome data as denominators for each row. 95% CIs for proportions were calculated using the Wilson method. ^†^Current practice in the home prefecture, practice in medically underserved areas, and primary care-related specialties were assessed at the survey time point.

In the best-case sensitivity analyses, in which missing outcome information was assumed to be present, estimates were modestly higher than in the main analysis. However, differences between waves and the overall interpretation remained consistent (Supplementary Table 1). The largest difference was observed for primary care-related specialties after the obligation period in 2021, where the best-case estimate was 80.0% compared with 62.0% in the main analysis.

## Discussion

The present study of JMU’s contract-based system showed consistently high compliance with the service obligation over approximately 45 years, with approximately 97% of graduates fulfilling the requirement. During the 9-year obligation period, graduates mainly practiced in their home prefecture, and after the obligation period, approximately 70% continued to work in the same prefecture. The JMU model appeared to realize high fulfillment of the service obligation and continued post-obligation practice in the home prefecture, even amid changing demographic, health-system, and societal contexts over time.

While the findings of the present study are consistent with prior studies reporting high compliance with service obligation among JMU graduates ^(3), (4)^, it is relevant to update and extend the evidence to demonstrate sustained retention with participation in primary care-related specialties over long-term follow-up. The present study did not examine the detailed data on the determinants of retention, but the JMU model includes the selection of students from rural backgrounds and meaningful rural training exposure during medical school ^(2), (8)^. In this context, the findings may provide a practical benchmark for post-obligation continued practice in the home prefecture when designing or evaluating scholarship-linked service programs in Japan, such as the regional quota program, while acknowledging differences in program rules and placement policies among the programs.

Knowing why continued practice in the same prefecture decreased after the obligation period is important. One possible explanation is that some physicians moved for specialist training and board certification. In Japan, board certification is linked to increased physician mobility, which often involves relocation to more urban areas ^(9), (10), (11)^.

Primary care-related specialties are thought to be a basis for working in medically underserved areas ^(3)^. The findings of the present study revealed substantial primary care participation across the obligation and post-obligation periods. For primary care-related specialties after the obligation period, the sensitivity analysis showed that the estimate could be meaningfully higher than in the main analysis in 2021. Under these assumptions, the true proportion lies between the main analysis estimate and the best-case estimate. Overall, these findings remain consistent with earlier studies showing that programs designed to address underserved workforce needs often achieve their impact through an orientation to primary care ^(12), (13), (14), (15), (16)^.

The present study had several limitations. First, we did not examine individual- or system-level determinants of retention. Second, measurements at three time points may not capture interim moves or the timing of attrition between waves. In addition, for the period-based outcome, the assessed period differed across some individuals, particularly among physicians surveyed during the obligation period. For outcomes assessed at the survey time point, residual confounding by career stage related to differences in years since graduation cannot be excluded. This may have been partly mitigated by the inclusion of newly graduated physicians in each wave. Third, medically underserved areas were classified using the 2021 designation and applied uniformly to the 2011 and 2016 survey waves. This may have introduced misclassification bias and affected temporal comparisons because regional physician distribution and healthcare needs may have changed over time. Fourth, generalizability may be limited because the JMU model was specifically established in Japan’s prefecture-based workforce governance and service obligation system. Finally, this study had no comparison group. While comparative evaluations during the obligation period have been reported, additional long-term comparative research is needed to clarify post-obligation outcomes ^(4)^.

## Conclusions

The proportions of JMU graduates practicing in their home prefecture and in medically underserved areas remained stable across survey years. Additionally, JMU graduates contributed substantially to nationally designated medically underserved areas and primary care. These findings may inform medical schools and policymakers in Japan and other countries when establishing contract-based rural training programs similar to the JMU model.

## Article Information

### Author Contributions

Conceptualization, Jun Watanabe and Kazuhiko Kotani. Methodology: Jun Watanabe, Akihisa Nakamura, Daisuke Matsubara, and Kazuhiko Kotani. Software: Jun Watanabe. Validation: Jun Watanabe and Akihisa Nakamura. Formal Analysis: Jun Watanabe. Investigation: Jun Watanabe, Akihisa Nakamura, and Kazuhiko Kotani. Resources: N/A; Data Curation: Jun Watanabe and Kazuhiko Kotani. Writing – Original Draft Preparation: Jun Watanabe. Writing – Review and Editing: Akihisa Nakamura, Daisuke Matsubara, and Kazuhiko Kotani. Visualization, Jun Watanabe. Supervision: Kazuhiko Kotani. Project Administration: Kazuhiko Kotani. Funding Acquisition, N/A.

### Conflicts of Interest

None

### Institutional Review Board Statement

This study was approved by the Ethics Review Board at Jichi Medical University (approval No. Clin 25-039). All the procedures were conducted anonymously in accordance with the Declaration of Helsinki.

### Data Availability Statement

The datasets analyzed in this study are available from the corresponding author upon reasonable request.

## Supplement

Supplementary Material
