# Supplementary material for: A Contract-based System for Physician Practice in Their Home Region in Japan: Follow-up Data of Graduates from Jichi Medical University (1978-2021)
Source: JMA J. 2026 Aug 28;9(5):1151–5. doi: 10.31662/jmaj.2026-0078 (PMC13639653; doi:10.31662/jmaj.2026-0078)
Supplement: Supplementary file 1 [file 2433-3298-9-5-1151-s001.pdf]

Supplement Table 1. Best-case Sensitivity Analysis (Missing Counted as Outcome-present) for All Graduates, During Obligation, and After Obligation.

Year	2011	% (95% CI)*	2016	% (95% CI)*	2021	% (95% CI)*
All eligible graduates, n†	3,441		3,963		4,534	
Practice in medically underserved areas, n	631	18.3 (17.1-19.7)	683	17.2 (16.1-18.4)	760	16.8 (15.7-17.9)
Primary care-related specialties, n	2,798	81.3 (80.0-82.6)	3,010	76.0 (74.6-77.3)	3,492	77.0 (75.8-78.2)
Eligible graduates after obligation, n†	2,548		3,028		3,511	
Practice in medically underserved areas, n	354	13.9 (12.6-15.3)	386	12.7 (11.6-14.0)	444	12.6 (11.6-13.8)
Primary care-related specialties, n	2,036	79.9 (78.3-81.4)	2,399	79.2 (77.7-80.6)	2,809	80 (78.7-81.3)
Eligible graduates during obligation, n†	695		712		787	
Practice in medically underserved areas, n	272	39.1 (35.6-42.8)	294	41.3 (37.7-44.9)	314	39.9 (36.5-43.4)
Primary care-related specialties, n	576	82.9 (79.9-85.5)	611	85.8 (83.1-88.2)	683	86.8 (84.2-89.0)

CI, confidence interval.

*Percentages were calculated using eligible graduates with non-missing outcome data as denominators for each row. 95% confidence intervals (CIs) for proportions were calculated using the Wilson method.

†"All eligible graduates" refers to the full eligible cohort. "Eligible graduates during obligation" refers to graduates in postgraduate years 3–9, and "eligible graduates after obligation" refers to graduates in postgraduate years ≥10.
